# Health care professionals’ knowledge, attitudes and practices relating to umbilical cord blood banking and donation: an integrative review

**DOI:** 10.1186/s12884-016-0863-6

**Published:** 2016-04-19

**Authors:** Lisa Peberdy, Jeanine Young, Lauren Kearney

**Affiliations:** University of the Sunshine Coast, Sippy Downs, QLD Australia

**Keywords:** Cord blood banking, Health care professionals, Knowledge, Opinions, Practices, Antenatal education

## Abstract

**Background:**

Collection and storage of an infant’s cord blood at birth is an option available to many new parents. Antenatal health care providers have an important role in providing non-biased and evidence based information to expectant parents about cord blood and tissue banking options. The aim of this paper was to identify and review studies of health care professionals’ knowledge, attitudes and practices concerning cord blood banking and the sources by which healthcare professionals obtained their information on this topic.

**Methods:**

An integrative review was conducted using several electronic databases to identify papers on health care professionals’ knowledge, attitudes and practices pertaining to cord blood banking. The CASP tool was used to determine validity and quality of the studies included in the review.

**Results:**

The search of the international literature identified nine papers which met review inclusion criteria. The literature review identified that there was little focus placed on antenatal health care professionals’ knowledge of cord blood banking options despite these health care professionals being identified by expectant parents as their preferred, key source of information.

**Conclusion:**

Limited high quality studies have investigated what health care professionals know and communicate to expectant parents regarding cord blood banking. Further research should focus on understanding the knowledge, attitudes and practices of healthcare professionals and how they communicate with expectant parents about this issue. In addition, how this knowledge influences professional practice around birth is also important, as this may positively or negatively impact the information that is provided to expectant parents.

## Background

Parents today have the option to donate or to privately bank their infant’s umbilical cord blood for use at a later date if therapeutic need arises. How parents come to make this decision is multi-factorial, and arguably significantly influenced by those caring for them during the antenatal period. This paper presents a discussion regarding umbilical cord blood banking and donation with a review of the published literature addressing the knowledge, attitudes and practices of health care professionals involved with pregnant women and their families during the perinatal period.

Umbilical cord blood banking is the process of collecting and storing umbilical cord blood, in the immediate period after the birth of a baby [1]. Umbilical cord tissue banking is the process of collecting and storing a small segment of the umbilical cord after the delivery of the placenta [2].

For the past 25 years cord blood has been used as an alternative to bone marrow for treatment of blood, immune system and metabolic disorders because of its rich source of haematopoietic stem cells [3]. Cord blood stem cell transplants are now an approved therapy for over 80 medical conditions. Cord tissue is a rich source of mesenchymal stem cells which show great potential for use in regenerative medicine. There are many clinical trials underway investigating the benefit of haematopoietic and mesenchymal stem cells for neurological and autoimmune disorders such as Cerebral Palsy, Autism and Type 1 Diabetes [4, 5].

As a result, cord blood banking is a growing phenomenon, with an increasing number of cord blood units being collected and stored [6]. The first public cord blood bank opened in New York in 1993 [7–10]. Currently, an international network of 158 public cord blood banks in 36 countries, houses over 731 000 umbilical cord blood units [11–15]. To date, public cord blood banks do not collect and store cord tissue. Private cord blood banking has had the most rapid rise in uptake [6]. There are currently 207 private cord blood banks which have been established in 54 countries, marketing services to a further 35 countries [11]. Over one million cord blood units have been stored privately and the growth rate is estimated to be approximately 12–15 % per annum [16, 17]. Since 2008, many private banks also collect and store cord tissue.

Public cord blood banks collect, process, test, store and release cord blood units which have been altruistically donated for allogeneic use, at no cost to the donating parents [18]. Allogeneic transplants use donated stem cells from another person who is genetically matched. Donors may or may not be related to the transplant recipient.

Private cord blood banks charge a fee to collect, process and store an infant’s cord blood for autologous or allogeneic family use [18–22]. Autologous transplants use one’s own stem cells.

For expectant parents, the decision to donate or privately store cord blood is a personal one [23]. An increasing number of expectant parents are seeking information from their antenatal care provider about cord blood banking options [9, 24–27], or requesting their assistance in private cord blood collection [25, 28, 29]. Internationally, studies have been conducted investigating pregnant women’s and/or expectant parents’ knowledge and perceptions of cord blood banking. Results from multiple studies have revealed that the majority of respondents would like to receive information regarding cord blood banking and donation from their antenatal care provider [30–36]. Antenatal care providers have an important role in providing accurate, unbiased, evidence-based information about cord blood banking options to assist expectant parents with their decision [1, 22, 27, 37, 38]. Some countries have acknowledged this important role by introducing legislation that recommends or requires healthcare professionals to inform expectant parents of their options concerning cord blood preservation [39]. However it has been suggested that private cord blood banks are frequently the main education sources about cord blood banking for health care professionals, including obstetricians [40], which raises ethical issues about the quality of unbiased information subsequently imparted to parents. In addition, midwives and midwifery professional groups have raised concerns about the added burden that cord blood collection places on midwives during the critical period of placental delivery when it is proposed that the focus of care should be on the mother and her newborn, particularly in resource limited environments [29].

It is therefore timely that research into healthcare professionals’ knowledge, attitudes and practices concerning the collection and storage of cord blood and tissue is explored to identify gaps which exist in knowledge that may influence health professional attitudes and practices towards informing expectant parents about their options. Despite the option to privately bank cord tissue, no studies to date have investigated health care professional knowledge, attitudes and practices regarding this; therefore, for the purpose of this review, only cord blood banking will be discussed.

## Methods

### Aim

This integrative review explored the research question ‘What knowledge, attitudes and practices do maternity healthcare professionals have relating to cord blood banking and donation?’ Specific aims of this review were to identify and critique a) studies of maternity healthcare professionals’ knowledge, attitudes and practices concerning cord blood banking and donation, and b) the sources by which these healthcare professionals obtained their information regarding cord blood banking and donation.

### Methodology

The integrative review method was chosen as this approach allows for a combination of diverse methodologies to be reviewed [41] and allows for the rigorous evaluation of the strength of the evidence, identification of gaps in the literature and the need for further research that provides a contribution to the topic [42]. The framework guiding this integrative review was based on Whittmore and Knafi’s proposed five stages model [41]; problem identification, literature search, data evaluation, data analysis, and presentation [42].

**Fig. 1:**
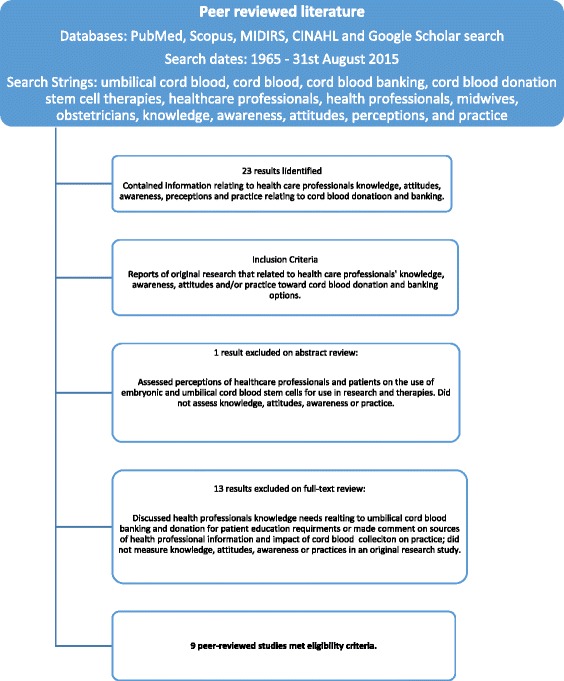
Screening and inclusion process

Figure 1 details the structured search conducted, including search strategy and inclusion process applied to the peer reviewed literature which was included in this integrative review. This integrative review aimed to identify all available original studies of health care professionals’ knowledge, attitudes and practices of cord blood banking and donation, and how these factors have been previously described. Publication dates were therefore inclusive of literature published between 1965 and August 2015 and no studies were excluded based on poor study quality. Due to resource limitations, articles were limited to those available with an English translation. The first author conducted the initial search and identified the potential papers for inclusion based on their title and abstract, with all papers for inclusion and exclusion discussed and agreed upon by all authors. Ethical review was not required for data accessed and included in this integrative review due to its availability in the published literature.

### Data analysis

To identify health care professionals’ knowledge, attitudes, practices and sources of information pertaining to umbilical cord blood banking and donation, each article was read and summarised to identify the key points. The papers were grouped using professional categories of maternity nurses, midwives and obstetricians. This review included staff who directly provided antenatal care including information to parents (e.g., maternity nurses, midwives and obstetricians). One study (Hatzistilli et al. 2014) meeting eligibility criteria [43] also included a small number of laboratory and administrative staff (mostly nurses) in the health professional sample (7 of 109 participants). As results were presented as combined health professional responses, this study was included in the integrative review. Studies which included maternity nurses or midwives described that both groups provided care for women in the antenatal, intrapartum and postpartum period of childbirth [27, 43–47]. However for the purpose of this review maternity nurses and midwives were analysed separately due to the potential for different service models and educational requirements apparent in developed and developing countries. Following identification of common themes, similarities and differences between these three professional groups (obstetricians; midwives and maternity nurses) were compared. Critical Appraisal Skills Programme (CASP) tools appropriate for the study design were used to determine validity and quality of the studies [48]. Each CASP Tool considers three broad issues when appraising studies: Are the results of the study valid? What are the results? Will the results help locally? [48]. The quantitative studies were assessed using the CASP Cohort Study Checklist. The qualitative/mixed methods studies were assessed using the CASP Qualitative Checklist.

## Results

Nine papers of health care professionals’ knowledge, attitudes and practices pertaining to cord blood banking and donation met review inclusion criteria. Although the content of two discussion papers [29, 40] addressed key aims relating to health professional knowledge and education, sources of information and current issues faced in practice, these were excluded from final review as they did not meet the criteria for original research. Empirical studies selected for this review used qualitative (*n* = 1), quantitative (*n* = 5) and mixed methodologies (*n* = 3).

Papers regarding health care professionals’ knowledge, attitudes and practices pertaining to cord blood banking and donation were conducted in North America [49], the United Kingdom [27, 46], Europe [43, 47], North Africa [44, 45] and Asia [50, 51]. No studies were located within Australasia or South America.

The integrative review included descriptive quantitative studies predominantly using survey designs [43, 44, 49–51] or a mixed method approach (survey design combined with semi-structured interviews and/or focus groups to yield qualitative data) [45, 46, 50, 51] to describe health care professionals’ knowledge, attitudes and practices pertaining to cord blood banking and donation (Please see Table 1).

**Table 1:** Overview of papers included in the review

Author/Year	Aim	Country/setting	Sample/Inclusion	Design and Methods/Tools	Results	Limitations
Duffy et al. (2009)	To assess attitudes of health professionals, explore perspectives, knowledge and experiences of donors, and analyse quality and risk of collection methods of umbilical cord blood for donation purposes. To examine pre and post-test responses following introduction of the Kingscord cord blood training.	England Acute teaching hospital with maternity and cord blood collection and donation service	Midwives Target = 150 Pre-test = 59 (40 % response rate) Post-test = 47 (31 % RR total; 79.7 % pretest)	Quantitative Survey: Pre and post education	Findings reported from midwifery participants only. Overall: Enthusiasm for cord blood collection; Concerns regarding staffing as a potential problem. Posttest: useful education with improved knowledge of conditions that could benefit from public CB bank Pretest: *Knowledge* *All participants aware of CB use for haematological disorders. *66 % (*n* = 39) aware additional uses: autoimmune, genetic and degenerative diseases/conditions. *44 % (*n* = 26) wanted more information. *Of 23 who had CB collection experience (39 %), 14/23 (86 %) had performed cord blood collection without prior training. *Attitudes* *Positive responses to program good to fantastic (55, 93 %) *4 (6.7 %) midwives expressed concerns regarding extra paperwork associated with cord blood donation/collection. *Posttest: *Knowledge* *Training sessions useful in understanding whole process (29, 62 %) rationale to collect (9, 19 %) and some practical details (9,19 %). *Attitude* *94 % (*n* = 44) indicated training was very useful; answered all questions *66 % (*n* = 31) stated collection training provided all necessary information. *Participants stated they learned more about cord blood treatable conditions. *19.1 % suggested the introduction of support in practice.	Small sample size with low response rate for target population (40 % pretest, 31 % posttest) Survey items and composition not discussed in detail and posttest did not appear to replicate pretest. Nil validity or reliability testing reported Unable to determine if primary aim of assessing attitudes of HP was achieved.
Salvaterra et al. (2010)	To analyze knowledge, comprehension, opinions, attitudes and choices related to cord blood donation of pregnant women, future parents, donors, midwives, obstetricians/gynaecologists. To compare preferences of public versus private banking.	Italy Hospital, community & academic sector participation	Antenatal health care providers (*n* = 32): Community midwives (*n* = 10) Hospital midwives (*n* = 10) Obstetricians (*n* = 10) Pregnant women, future parents and donors (*n* = 30)	Mixed methods using participatory approach with establishment of a taskforce and public multidisciplinary round table Focus groups; (maximum 10 participants, led by 2 psychologists) Survey: Anonymous self -administered (Demographic, knowledge, opinion items)	Findings reported from midwives and obstetrician/gynaecologists only. Overall: Support for altruistic cord blood donation; Better health professional education needed. Focus Group Themes: *Hospital & community midwives recognised social/moral value of CB donation Vs no social/moral value recognition by obstetricians *100 % hospital midwives expressed a negative moral assessment of private cord blood banking *Midwives viewed obstetricians as providing crucial role in providing parents with information *All disciplines agreed obstetricians can promote CB donation through providing accurate information to expectant parents. *Specific education and HP support roles needed to promote donation Questionnaire *Obstetricians (30, 94 %) & midwives (28, 87.5 %) were preferred providers of information to parents *Information should be provided in prenatal courses (24, 75 %) *Altruistic (25, 78 %) and moral duty (7,22 %) were factors in supporting public donation Outcome: Development of information brochure	Few knowledge questions; most opinion based. Small sample sizes allowed for limited between group comparisons Researchers developed own assessment tool, nil validity or reliability testing reported
Tada et al. (2011)	To understand how obstetricians involved in cord blood collection view and think about this collection process.	Japan Four Public Cord Blood Banks in two metropolitan areas.	Hospital representative obstetricians who were involved in cord blood collection activities. Questionnaire (*n* = 38) Semi-structured interviews (*n* = 8)	Qualitative/Mixed methods Survey: Self-administered Semi-structured interviews	Overall: Obstetricians generally willing to participate in cord blood collection. *Status of CB collection* * 60.5 % indicated there was no CB collection training course at their institution. * 28.9 % believed that CB collection training is necessary prior to collecting CB. 68.4 % believed that CB collection in addition to their routine care did not place a burden on them. * 84.2 % believed that CB collection did not pose a risk prior to or following delivery. *Training courses* * 6 respondents answered that if CB is simply collected, a training course is not mandatory. However, a training course prior to collecting CB would assist to collect greater volumes and prevent bacterial contamination. *Burden and risks associated with CB collection* *All respondents agreed that there were no risks or burdens as CB collection is stopped if anything goes wrong at the time.	Small sample of a large number of obstetricians in the 53 hospital settings. Results represent obstetricians’ beliefs and opinions, not knowledge. Assessment tool adapted from tools used in previous cord blood stem cell quality studies. Ethical approval not disclosed.
Machin et al. (2012)	To explore the perceptions of key stakeholders (professional and lay) in cord blood banking relating to the role of midwives and privately employed phlebotomists.	England Hospital, community, CB banking, academic and government sector participation	Purposive sample of stakeholders in UCB Banking (total *n* = 69) Midwives (*n* = 15) Obstetricians (*n* = 8) Neonatologists (*n* = 2) CB Banks (*n* = 12) Phlebotomists/donor coordinators (*n* = 3) Policy and Government representatives (*n* = 4) Royal Colleges (*n* = 2) Activist, genetic interest and professional groups (*n* = 9) Scientists (*n* = 4) CB bank clients (parents) (*n* = 10)	Qualitative Semi-structured interviews (*n* = 61) including Midwives (*n* = 7); Obstetricians/neonatologists (*n* = 10); Other (*n* = 44) Focus Group: 8 midwives	Overall: 3 key themes identified as Negotiating space; Negotiating access and Negotiating priorities and practices. Acknowledgement of midwives as gatekeepers in access to umbilical cord blood: themes of power, authority and control. Results highlighted: *Midwives were perceived as regarding privately employed phlebotomists/CB collectors as external to woman’s care team *Perception that midwifery practice is prioritised over request for cord blood collection as it benefits maternal and infant health and wellbeing, while CB collection seen as a non-essential ‘option’ or ‘wish’ than can alter ‘important’ midwifery practice. *Perception that Midwives rank CB collection for private banking as a low priority. *In contrast, Perception of pregnant women that midwives are in a less powerful role than pregnant women relating to UCB collection (i.e. women’s wishes are the priority), supported by midwifery responses.	Member checking in interpretation of qualitative data was not reported; limits content validity relating to interpretation of midwifery roles and priorities.
Walker et al. (2012)	To measure obstetricians’ levels of awareness and understanding of CB donation and CB therapy; To measure obstetricians motivation to support CB donation and collection; To determine frequency of discussion of CB banking options with patients.	United States 57 Metropolitan hospitals affiliated with a public CB bank	Obstetricians (target *n* = 2041) Total (*n* = 295), 14 % response rate Obstetricians with privileges at affiliated hospitals (*n* = 139) Obstetricians without privileges at hospitals affiliated with public cord blood banks (*n* = 156)	Quantitative Survey: Multi-choice question, 1 open ended question	Overall: Obstetricians are generally familiar with cord blood transplantation though indicated a desire for more information to effectively inform patients. Similar findings between obstetricians affiliated with a CB donation hospital versus a nonaffiliated hospital. *Understanding of CB Therapy* *88 % of affiliated and 82 % of non-affiliated obstetricians (*n* = 122/128) reported being familiar or very familiar with cord blood transplant use. *Source of information* *87 % of affiliated and 84 % of non-affiliated obstetricians (*n* = 121/131) indicated private CB banks as their main source of information *Awareness of CB donation* *98 % of affiliated and 96 % of non-affiliated obstetricians (*n* = 136/150) knew that private CB banks charge collection and storage fees. *86 % of affiliated and 69 % of non-affiliated obstetricians (*n* = 119/108) knew that there were no associated fees for women to donate CB. *Willingness to discuss CB donation with patients* *80 % of affiliated and 70 % of non-affiliated obstetricians (*n* = 111/109) were confident discussing CB banking options with patients. *49 % (68/139) of affiliated and 51 % (79/156) of non-affiliated obstetricians felt they had insufficient knowledge of CB donation to effectively answer patient questions. *Willingness to collect CB and perceived barriers to CB collection* *36 % of affiliated and 35 % of non-affiliated obstetricians (*n* = 50/55) agreed that lack of compensation for doctors is a barrier to public CB collection. *Most common response to patient question regarding what she should do with CB* *37 % of affiliated and 41 % of non-affiliated obstetricians (*n* = 51/64) stated they discuss all options and benefits and let patient decide.	Survey tool developed and pretested by the National Bone Marrow Program specific for this study. Separate surveys used for affiliated and non-affiliated participants.
Hatzistilli et al. (2014)	To investigate health care professionals’ knowledge, attitudes and sources of information on UCB donation.	Greece 5 Hospitals: 2 urban (1 with CB bank and transplant centre), 3 rural general hospitals	Midwives (*n* = 47) Obstetricians/Anaesthetists (*n* =28) Nurses (*n* = 29) Administration nurses (*n* = 26) Response rate = 84 % (*n* = 109/130)	Quantitative Survey: Yes/no: knowledge level Self-rating question: information sources. Internal consistency determined (Cronbach Alpha 0.73)	Overall: The study highlighted low levels of informed HP knowledge (15.6 %) and willingness to participate in well organized continuous education for specialists (89 %). *CB donation knowledge* *15.6 % reported as well informed on collection, storage and use of UCB *Correct responses mean 55.2 % (SD ± 18.5); median 57 % (IQ 25) *93.5 % declared little or no specific UCB education in last 5 years *Obstetricians had significantly greater knowledge levels (62.6 %) compared to midwives (59 %) and other health professionals (*p* < 0.002)relating to cord blood collection, donation, private storage, quality and therapies. *Sources of information*: *Promotional brochures (33 %), * private CB banks information(22 %), *magazines and newspapers (19.2 %), *bachelor/master academic levels and scientific brochures (18.4 %), *seminar and conferences (13.7 %) *National Organisation of Transplantations (EOM) Website (11 %)	Small sample size, limiting generalisability. 1 participating hospital had a cord blood bank. Researchers developed own questionnaire (internal consistency measured)
Roh et al. (2014)	To investigate obstetricians understanding of CB – collection, legal regulations, limitations and potency of CB banks, and current therapeutic uses - and their role in informing donors and cord blood collection management. Investigate the quantity of cord blood information provided to patients by obstetricians.	Korea CB collection centres associated with public CB banks	Obstetricians (*n* = 57) Representing CB collection centres (*n* = 32) with cord blood collection experience.	Quantitative Survey: Multi-choice: self-rating knowledge assessment.	Overall: Obstetricians comprehensive understanding of CB banking, collection, transportation, storage, regulations, therapeutic uses and limitations, and potency of public cord blood banking were lower than expected. *CB collection experience and management* *82.5 % had a minimum of 4 years collection experience. *40.4 % were aware that CB collection was regulated by law. *21 % felt that CB collection distracted the labour process. *15.8 % were concerned about delayed bleeding control during CB collection especially during LSCS delivery. *Knowledge about CB current uses* *82.7 % correctly rated the therapeutic uses of cord blood. *54 % self-rated their knowledge level about cord blood usefulness as average. 30 % self-rated their knowledge as below average. *Response to patient requests made for information on difference between public and private CB banks* *5.3 % did not provide any information. *61.4 % provided contact information for public and private banks. *33.3 % verbally informed women of the differences between the banks. (100 % of this group showed the highest rate of CB processing post collection.)	A small sample of a large number of cord blood collection centres so results may not be generalised to the population. Survey tool adapted from Walker et al. (2012) study.
Mohammed & El Sayed (2015)	To evaluate the effectiveness of educational program on maternity nurses’ knowledge and attitude regarding cord blood collection and stem cells.	Egypt 2 hospital settings: labour unit, University hospital and maternity University hospital	All maternity nurses employed in the 2 settings at time of study were included. Total (*n* = 53) Obstetric department (*n* = 28) Maternity hospital (*n* = 25)	Pilot with content validity by expert panel, Reliability testing Cronbach’s Alpha for Knowledge =0.92, Attitude 0.87. Survey: Pre and post education: closed and open-ended questions. Part 1: Knowledge and Demographics. .Part 2: Attitudes: Likert Scale, Pretest survey results informed education intervention Post-test survey: 1)immediately post education 2) 3 month post education	Overall: Maternity nurses’ knowledge and attitudes towards CB collection and stem cells improved following education intervention. Knowledge *Pre-education: 88.7 % had poor knowledge levels of cord blood collection and stem cells. *Post education: 90.6 % had good knowledge levels of cord blood collection and stem cells immediately post education; 81.2 % good knowledge at 3 months post education. Attitudes *Pre-education: 98.1 % of had a negative attitude towards cord blood collection and stem cells. *Post education: 66 % had a positive attitude to cord blood collection.	Educational session duration not consistent due to workload management. Educational intervention developed specific to participant areas and processes, limited use in other countries dependent on content.
Moustafa & Youness (2015)	To assess maternity nurses knowledge of cord blood banking and to identify barriers of it to be applied.	Egypt Large Obstetrics & Gynaecology Department in University Hospital	Convenience sample of maternity nurses employed in antenatal, labour, postnatal wards and antenatal clinic (*n* = 150).	Quantitative Survey: Descriptive Design of demographics and knowledge Content Face validity established through use of expert panel and pilot.	Overall: Knowledge about CB banking was reported as inadequate. Barriers to CB banking were identified as cost of CB banking, policies and procedures of conducting new technology at the hospital. *Main Source of information* *Books & magazines (39.3 %), seminars & conferences (14 %). *Level of knowledge* *78.7 % of nurses reported as having inadequate knowledge of cord blood banking. *58 % reported they did not know the advantages of CB banking & 34 % did not know the disadvantages of CB banking. *14 % did not know CB collection procedures. *95.3 % wanted further information through training and education programs *More experienced and qualified nurses were more likely to have adequate knowledge scores *Barriers to providing CB banking* *70 % believed cost of CB banks, followed by policies & procedures of hospital (66 %), time taken to educate women (54 %), lack of nurses knowledge & cultural beliefs (both 52 %), pregnancy not being suitable time to make decisions (38 %), lack of women’s knowledge (34 %), religious barriers (32.6 %).	Sample from one hospital, results potentially not generalisable across settings and other countries. Response rate not reported. Tool reported to have confirmed validity through use in other studies; no references provided for these other studies.

*CB* cord blood

Exploration included obstetrician perspectives, knowledge, awareness and acceptance of cord blood collection, donation, banking and use [49–51]; maternity nurses or midwives’ knowledge, awareness and attitude towards collection of cord blood for private banking and donation [27, 44–46]; and two multidisciplinary studies of obstetricians’ and midwives’ attitudes and opinions of cord blood banking and donation [43, 47]. Review findings are described from the perspective of maternity nurses, midwives and obstetricians as the majority of the studies reviewed (*n* = 9) examined these professional groups separately. Findings are discussed under three broad headings: knowledge, attitudes and practice.

### Knowledge

#### Midwives and maternity nurses

##### Knowledge and awareness

Four studies examined either midwives’ or maternity nurses’ knowledge and awareness of potential benefits and uses of cord blood stem cells.

Duffy and colleagues [27] evaluated the attitudes of midwives in relation to the impact of cord blood collection for donation in a large maternity unit. Findings revealed that midwives were aware that cord blood was used in the treatment of haematological disorders but only two thirds (*n* = 39) were aware of other indications for use such as autoimmune disorders, degenerative conditions and genetic diseases [27]. Hatzistilli and colleagues [43] assessed health professional knowledge regarding cord blood donation and reported that just over half the midwives surveyed (*n* = 22, 59 %) could provide correct responses regarding the collection, storage and use of cord blood. Moustafa and Youness [44] reported that most maternity nurses (*n* = 118, 78.7 %) in their study had inadequate knowledge of cord blood banking [44]. Mohammed and El Sayed [45] assessed maternity nurses’ knowledge of cord blood, collection and uses before staff participated in a directed education program regarding cord blood. The authors found that most participants (*n* = 47, 88.7 %) had poor knowledge of cord blood, collection and uses. Immediately following the education program and completing the same questionnaire, maternity nurses’ knowledge increased, with the authors reporting that 90.6 % (*n* = 48) of the participants had a good knowledge of cord blood, collection and uses, and this was sustained at 3 months post education [45].

##### Source of information

Hatzisilli and colleagues [43] reported that of 109 antenatal healthcare professionals, most (*n* = 102, 93.5 %) had little or no formal education on cord blood collection, storage options and transplantation within the past 5 years [43]. Similarly, Mohammed and El Sayed [45] revealed 98.8 % (*n* = 52) of the maternity nurses had not attended cord blood collection and stem cell training courses [45].

Hatzistilli and colleagues [43] identified the main source of information on cord blood and banking for midwives was provided by private cord blood banks through clinic brochures and media promotion. Non-scientific magazines, newspapers, university courses, academic papers, seminars and conferences were also reported as cord blood banking information sources [43]. Moustafa & Youness [44] reported similar sources of information from their study of maternity nurses with the main sources being books and magazines (39.3 %) followed by seminars and conferences (14 %) [44].

##### Desire for more information

Several studies examined midwives and maternity nurses’ requirement for further information about cord blood collection and banking [27, 44, 47]. Midwives expressed a desire to receive information on private cord blood banking and use of cord blood, and information on cord blood collection steps and processes were sought by midwives prior to engaging in cord blood collection activities [27]. Moustafa and Youness [44] also reported similar results for participant maternity nurses (95.3 %) who expressed a desire for further information on cord blood banking through training courses and educational programs [44].

#### Obstetricians

##### Knowledge and awareness

Walker and colleagues [49] surveyed obstetricians’ knowledge levels and understanding, motivation to support cord blood collection for donation, and practices relating to patient discussions about donation and private banking options at donation and non-donation hospitals in the USA. Most obstetricians self-reported a good understanding of cord blood transplantation and the differences between donation and private banking of cord blood (>80 %), in addition to confidence in discussing options with families (>70 %) [49]. However, many reported less confidence in their ability to answer specific questions with half of the total sample (*n* = 147/295, 50 %) reporting they had insufficient knowledge of cord blood donation to effectively answer patient/family questions, regardless of public or private hospital affiliation [49].

Roh and colleagues [51] reported similar findings in a survey of obstetricians employed at cord blood donation hospitals in Korea. Obstetrician understanding of cord blood collection, transplantation, storage, legalities, current uses, limitations and potency of cord blood units was lower than expected with less than half aware that cord blood collection was regulated by law [51]. Despite most obstetricians (*n* = 47/57, 82.7 %) correctly rating the therapeutic use of cord blood, confidence in application of this knowledge was lower with most self-rating their knowledge about cord blood as average (31, 54 %) or below average (*n* = 17, 30 %) [51].

##### Source of information

Despite cord blood advances being reported in scientific literature, the main source of information for obstetricians on cord blood banking and use was from private cord blood banks, consistent with findings from midwifery colleagues [43, 49]. Other reported sources of information include non-scientific magazines, newspapers, university courses, academic papers, seminars and conferences [43].

##### Desire for more information

Two studies reported that obstetricians indicated a desire for more information on cord blood banking [49] and cord blood donation so they could effectively inform expectant parents. The studies did not elaborate on desired mode of delivery of this information although Hatzistilli and colleagues [43] reported that most health care professionals (89 %) believed an organised, continuous education program on cord blood transplantation developments would be beneficial [43].

### Attitudes

#### Maternity nurses and midwives

##### Attitudes and perceptions

Midwives were found to be supportive of cord blood donation as they could see the associated social and moral aspects, although support did not extend to private cord blood banking [47]. This option was viewed by some of the midwives to be a ‘trendy, grim, useless and a selfish act’ [47]. In the early days of cord blood banking, midwives felt pressured to collect cord blood for private banking purposes. Collection, labelling, packaging and associated paperwork for private banking were seen as an added burden to the midwifery workload [27] and these activities were not seen as a priority of midwifery care [46]. Mohammed and El Sayed [45] assessed the attitude of maternity nurses and found that before implementation of a cord blood education program, 98.1 % (*n* = 52) of participants were graded to have a poor attitude towards cord blood collection and stem cells. Following cord blood education, this percentage decreased to 34 % (*n* = 18) [45].

Machin and colleagues [46] proposed that the primary role and responsibility of midwives was to the health and well-being of the mother and infant. Cord blood collection was not considered to be contributing towards this, and in fact was considered by some to alter midwifery practice that was for the benefit of mother and infant [46]. One midwife surveyed by Duffy and colleagues [27] expressed concern with the potential clinical implications regarding the timing of the cord clamping.

Machin and colleagues [46] reported qualitative findings from a stakeholder analysis and suggested that the midwifery profession is focused on pregnant women, labour and birth, therefore potentially positioning them as gatekeepers to both labour ward and cord blood. When expectant parents had decided to bank their infant’s cord blood and utilised a private cord blood collector for this purpose, midwives were only accommodating of their presence ‘as long as the collector was unobtrusive, compliant with midwife’s requests and respectful of midwives’ authority over the umbilical cord blood’ [46].

#### Obstetricians

##### Attitudes and perceptions

Unlike the midwives discussed by Machin and colleagues [46], the obstetricians surveyed by Tada and colleagues [50] were generally accommodating of cord blood collection. Over two thirds (*n* = 26, 68.4 %) reported that cord blood collection in addition to their routine obstetric care did not place pressure on them, with only a few participants indicating that the consent process and collection documentation was extra work and time consuming [50]. Roh and colleagues [51] reported that 21 % of obstetricians (*n* = 12/57) indicated that cord blood collection distracted the labour process, and 15.8 % (*n* = 9/57) were concerned about delayed bleeding control as a maternal risk, especially in caesarean deliveries. One obstetrician reported concerns about the risk to the baby, but did not specify details [51]. The majority of obstetricians surveyed by Tada and colleagues [50] (*n* = 32, 84 %) though believed cord blood collection was a safe procedure and did not pose a threat to women before or after delivery [50].

### Practices

#### Midwives and maternity nurses

##### Education role

No papers investigated the role of midwives and maternity nurses in patient education on cord blood banking, although two papers briefly acknowledged their key role in antenatal education [31]. Duffy and colleagues [27] outlined midwives’ responsibility for providing a large volume of information to pregnant women, often requiring explanations and discussions so that patients are able to make informed choices about their care [27]. Moustafa and Youness [44] also briefly examined the maternity nurse role in education for pregnant women. Over half of the study participants (*n* = 81, 54 %) found that the time required to educate antenatal women on cord blood banking was one of the barriers to providing this education [44].

##### Practices

In response to concerns about risk associated with cord blood collection [50], Duffy and colleagues [27] conducted a risk assessment to investigate if cord blood collection interfered with labour outcome. Three groups of cord blood collection methods were analyzed: in-utero (*n* = 25), ex-utero (*n* = 21), and no collection (*n* = 15). In-utero collection occurs after the birth of the baby but before the delivery of the placenta. Ex-utero collection occurs after the baby and the placenta have been delivered [47]. No difference was recorded between the three groups in maternal blood loss, timing of cord clamping to placental delivery, infant APGAR scores and time of discharge from labour ward [27].

#### Obstetricians

##### Education role

Most studies (*n* = 4/5) including obstetricians in their samples supported findings which highlighted the importance of cord blood education for obstetricians, to allow them to accurately inform and support their patients in the decision-making process regarding their cord blood banking options [43, 47, 49, 50]. Studies emphasised that obstetricians acknowledge their role, and that of other health professionals, in educating and informing women about cord blood banking using accurate information [47, 49], and the need for specific education programs for antenatal health care providers on this topic.

##### Study quality

CASP (Critical Appraisal Skills Programme) tools were used to assess the validity and quality of the papers included in the review [48] (Please see Tables 2 and 3).

**Table 2:** Appraisal of studies by study design using CASP tools

Quantitative studies (Cohort Study checklist)																					
	Are the results of the study valid?												What are the results?					Will the results help locally?			
Article no.	Did the study address a clearly focused issue?	Did the authors use appropriate methods to answer their question?	Was the cohort recruited in an acceptable way?	Was exposure measured to minimise bias?		Was outcome measured to minimise bias?		Have the authors identified all confounding factors?	Have they taken account of the confounding factors in the design and/or analysis?		Was follow up complete?		Are results presented transparently and precisely?		Are the results plausible?			Can the results be applied to the local population?		Do the results fit with other evidence?	Does this study have direct implications for practice?
1	Y	Y	Y	N		N		N	N		N		Y		Y			Y		Y	Y
2	Y	Y	Y	Y		N		N	Y		Y		Y		Y			Y		Y	Y
3	Y	Y	Y	N		Y		Y	Y		Y		Y		Y			Y		Y	Y
4	Y	Y	Y	Y		N		N	N		Y		Y		Y			Y		Y	Y
5	Y	Y	Y	Y		Y		Y	Y		Y		Y		Y			Y		Y	Y
6	Y	Y	Y	Y		Y		Y	Y		Y		Y		Y			Y		Y	Y
Qualitative studies																					
Article no. (ref)					Was there a clear statement of the aims of the research?	Is qualitative methodology appropriate?	Was design appropriate to address the aims?			Was the recruitment strategy appropriate to match aims?		Were the data collected in a way that addresses the research issue?		Has the relationship between researcher and participants been adequately considered?		Have ethical issues been taken into consideration?	Was the data analysis sufficiently rigorous?		Is there a clear statement of findings?		Is the research valuable?
7					Y	Y	Y			Y		Y		N		N	N		Y		Y
8					Y	Y	Y			Y		Y		N		Y	Y		Y		Y
9					Y	Y	Y			Y		Y		N		Y	Y		Y		Y

Y=Yes; N=No

**Table 3:** Appraisal of studies by study design using CASP tools

Key:		
Article Number	Quantitative Studies	Qualitative Studies
1	Duffy et al., 2009	7. Tada et al., 2011
2	Walker et al., 2012	8. Machin et al., 2012
3	Hatzistilli et al., 2014	9. Salvaterra et al., 2010
4	Roh et al., 2014	
5	Mohammed & El Sayed, 2015	
6	Mustafa & Youness, 2015	

All studies addressed a clearly focused question using an appropriate method although most were of small sample size. Inconsistencies in sampling, data collection, and measurement of identified variables were noted between studies. There was no standard validated tool used to measure knowledge, attitudes and/or practices relating to cord blood donation and storage, with most studies developing and using their own tool. There was also a lack of detail as to survey item composition or questions used for focus groups/interviews, and in how data was analysed, therefore replication of the studies would be difficult.

## Discussion

Table 4 provides a summary of key findings of this review and implications for further research.

**Table 4:** Key findings and implications for future research

Key findings
• Obstetricians, midwives and maternity nurses self-reported sound levels of knowledge of general cord blood usage and processes involved, however deficiencies were revealed once specific, more detailed information was required • Main information source was private cord blood bank company materials • Obstetricians, midwives and maternity nurses desired more evidence-based, non-biased information on CBB • Obstetricians, midwives and maternity nurses perceived an important role was to provide expectant parents with information to make informed decisions • Obstetricians held a positive attitude toward CBB and collection and perceived it required minimal extra work outside usual role. Many midwives and maternity nurses did not share this view, and perceived it as interfering with their primary role with some support for public, but not private, cord blood collection and storage
Implications for further research
• Health care professionals, such as obstetricians and midwives, are perceived to be the most credible providers of information regarding CB collection and storage, yet report their key information source as private cord blood collection company adverts and resources • Each study in this review utilised a different data collection instrument, which was often poorly described, yielding variant results, with little ability to compare between studies • Small, convenience samples were predominantly utilised in review studies minimising generalisability of findings • Knowledge tested was self-reported by participants • Urgent need to address knowledge deficiencies and explore underlying attitudes of health care providers, given vast advances occurring in stem cell research

Few studies (*n* = 9) have examined knowledge, awareness, attitudes and practices of obstetricians and midwives to cord blood banking and donation. Despite significant scientific advances, increases in clinical applications of cord blood stem cells, and availability of cord blood banking options for expectant parents over the last two decades, very little published work has reported on the knowledge and attitudes toward cord blood banking among healthcare professionals who care for pregnant women and their families.

Expectant parents identify health care professionals (obstetricians, midwives, maternity nurses) as the key source of information about cord blood banking options for expectant parents [21, 22, 37, 38, 52–54]. This review has shown that despite obstetricians, midwives and maternity nurses self-reporting that they have sound knowledge on the topic, there are differing levels of confidence in this knowledge and attitudes towards cord blood banking and donation reported by the professional groups. The provision of information to expectant parents on cord blood banking and donation, and the attitude towards cord blood collection vary among obstetricians, midwives and maternity nurses. The lack of formal education and training in cord blood stem cell uses, banking and donation has been highlighted, with private cord blood banks identified as the most common information source for health professionals. The use of private cord blood banking materials as the key source of information regarding cord blood stem cell collection and use is also concerning due to a conflict of interest.

Two studies showed that knowledge of obstetricians on this topic was reasonable although several areas were identified for improvement, including knowledge of collection process, regulation and implications for birth practices [49, 51]. In contrast, two other studies reported that most maternity nurses had low knowledge levels of cord blood and stem cell collection and uses [44, 45]. Few studies included multidisciplinary samples, however in one study which did, maternity nurses and midwives’ knowledge and awareness of cord blood uses appear proportionally lower then obstetricians (59 % vs 62.6 % respectively) [43]. Targeted education for maternity nurses relating to cord blood collection and use was demonstrated to be effective in achieving improved knowledge and attitudinal responses, when measured immediately post education, and over a sustained period [45].

The attitude and perceptions toward cord blood collection and banking varied between midwives and obstetricians. Midwives were supportive of the altruism behind cord blood donation although many could not see the advantage of private cord blood banking [47], with the collection process considered burdensome and interfering with midwifery care [27, 29, 46]. These findings suggest that midwives could be regarded as gatekeepers toward cord blood banking opportunities for expectant parents, highlighting an area for potential further research. One study demonstrated that targeted education could positively impact negative attitudes towards cord blood collection, with positive correlations between knowledge and attitude, and improved knowledge being associated with positive attitudes [45]. Obstetricians on the other hand were, overall, supportive of both public and private banking options [50, 51].

Despite different knowledge and attitudes of obstetricians, maternity nurses and midwives regarding cord blood banking, these professions requested further information on the topic [49], and many had not received recent formal education on cord blood collection, storage options and transplantation [43]. Most studies [43–45, 47, 49, 51] highlighted the importance of health care professionals being educated on cord blood banking so that they can accurately and confidently discuss this with their patients.

The studies reviewed in this paper demonstrated disparate results with little cohesion between research approaches and findings. This integrative review has revealed a paucity of studies on health care professionals’ knowledge, attitudes, practices and information sources of cord blood banking, identifying an area for future research. Education requirements for health care professionals on this topic need to be assessed to identify knowledge deficits. Results of such research studies could be used to inform evidence-based antenatal care provider education to facilitate accurate and effective discussions with expectant parents relating to cord blood banking and donation. This would assist expectant parents to make an informed choice on their option of cord blood and tissue banking based on the family’s values and needs.

### Strengths and limitations of this study

To the authors’ knowledge, this is the first review of the literature addressing cord blood banking and donation. This review is timely and relevant to clinical practice and parent education given the acknowledged importance of the role of health professionals in supporting parents in making informed decisions about their options based upon best available evidence [43, 47]. Reports from several continents and including samples from varied professional groups caring for families in maternity settings, with all reports published since 2009, reflects the increasing impact and relevance that cord blood donation and storage is having within health services around the globe.

Most studies in this review had relatively small samples sizes (*n* = 32 to 295); however frequently the small sample size was appropriate to the qualitative or mixed methods designs that were used [45–47, 50]. Survey tools to measure knowledge, attitudes and self-reported practices were often poorly described; few utilised previously used tools [50]; three studies reported attempts to establish validity and reliability of tools [41, 45, 48], with two of these studies reporting results of reliability testing [41, 47]. These limitations, together with varied service delivery models across several different countries, limits the generalisability of results across health settings, and highlights the need for studies that consistently use valid, reliable and well described tools with larger samples of health professionals, in order to reliably inform effective clinician education relating to cord blood storage and donation.

### Implications for healthcare professionals

Health care professionals are considered by the public to be the most credible source of information on cord blood banking [43]. This review has shown that to date there is little evidence to support this belief and further research into this topic is warranted to identify health care professionals’ knowledge and practices regarding cord blood banking. This will assist to develop strategies that optimally assist health professionals to perform this role.

To meet this public expectation, health care professionals need to have evidence-based knowledge on cord blood banking in order to provide expectant parents with accurate and un-biased information to facilitate informed choices regarding cord blood donation or private banking. Further research identifying areas for improvement in health care professional knowledge needs to be undertaken with valid and reliable tools. Translation of these research findings will inform the development of quality, tertiary and professional development education programs for midwifery and medical students on cord blood collection, banking and therapies to ensure consistency of curriculum across health care disciplines of antenatal health care providers.

### Implications for future research

Further research is required to identify and investigate health care professionals concerns regarding the practice of cord blood collection, the sources and influences associated with health professionals’ negative views about cord blood banking, timing of cord clamping and safety of mother and infant. Understanding these factors may assist in addressing health professional knowledge and attitude deficits [53], which in turn impact their ability to provide parents with evidence-based, unbiased information to support autonomous parental decision-making in this important area.

## Conclusions

This integrative review identified few studies of healthcare professionals’ sources of information, knowledge, attitudes and practices relating to cord blood banking. A considerable gap exists regarding understanding the information requirements of health professionals, and the influences on professional practice, as they relate to cord blood banking which impact on health professional provision of evidence-based information of cord blood banking options.

A key role of the healthcare professional caring for the expectant woman and her family is to impart unbiased, evidence based information in order to assist parents to make decisions about their care that best suits the needs of their family and reflects their own values, beliefs and priorities. Further research should focus on understanding the attitudes and opinions of health professionals, and how their birth management practices may be influenced by cord blood collection, as this may positively or negatively impact the information that is provided to expectant parents.
